# Case Report: Two atypical cases of M6-like antimitochondrial antibody pattern unlinked to iproniazid

**DOI:** 10.3389/fimmu.2026.1675688

**Published:** 2026-05-29

**Authors:** Elise Tolmer, Benjamin Bonnet, Gaelle Jeannin, Maud Junda, Marine Godignon, Bertrand Evrard

**Affiliations:** 1 Immunology Department, Clermont-Ferrand University Hospital, Clermont-Ferrand, France; 2 Immunology Laboratory, UNH UMR1019, University of Clermont Auvergne, INRAE, Clermont-Ferrand, France; 3 Thoracic Oncology Department, Clermont-Ferrand University Hospital, Clermont-Ferrand, France

**Keywords:** autoimmune hepatitis, indirect immunofluorescence, iproniazid, M6-subtype antimitochondrial antibody, pembrolizumab

## Abstract

Rare M6-subtype antimitochondrial antibody (AMA-M6) has been described in patients treated with iproniazid, an antidepressant drug. Diagnosis can be made by identifying its highly characteristic indirect immunofluorescence pattern on rat organ substrates. In the existing literature, the presence of AMA-M6 has been associated with hepatitis cytolysis, with a favorable outcome following the discontinuation of treatment. This report presents two cases of AMA-M6-like pattern in patients who had never taken iproniazid. In the first case, the patient was receiving pembrolizumab treatment for lung adenocarcinoma. The presence of this pattern was identified 20 days after introducing immunotherapy, with a diagnosis of liver cytolysis made 10 days later, associated with the presence of nodular regenerative hyperplasia. Following the suspension of the injection, liver enzyme levels decreased and the pembrolizumab treatment was resumed with no recurrence of liver damage. In the second case, the same AMA-M6-like pattern was discovered fortuitously in a patient presenting with a complaint of dry mouth only, and no evidence of liver damage. In contrast to previous reports, our findings do not support the presence of MAO-B–specific autoantibodies in these patients, and the target antigen remains to be identified. These two cases demonstrate that AMA-M6-like pattern can develop in patients who have never been exposed to iproniazid. While liver damage was present in one case, the patient remained asymptomatic in the second case. Immunologists must thus watch for uncommon patterns in indirect immunofluorescence to ensure appropriate medical care and patient follow-up.

## 1 Introduction

Antimitochondrial antibodies (AMAs) are a heterogeneous group of autoantibodies directed toward a variety of mitochondrial proteins. A total of nine distinct AMAs specificities have been identified in the serum of patients with various diseases, both liver-related and non-liver-related (1, 2). Among these, the M2 subtype (AMA-M2) is the most prevalent and best studied. AMA-M2 recognizes mostly the E2 subunit of pyruvate dehydrogenase complex (PDC-E2), an enzyme located in the mitochondrial matrix (3, 4). These autoantibodies are present in the serum of patients with primary biliary cholangitis (PBC), a chronic inflammatory autoimmune liver disease characterized by the obstruction and destruction of intrahepatic bile ducts, leading to fibrosis and cirrhosis (3, 5). Advances in molecular biology have allowed the identification of a wider range of AMAs, including the M6 subtype (AMA-M6).

AMA-M6 was first described by a French research team in 1981 (6). They reported a new immunofluorescence pattern, especially in rat organs (liver kidney stomach substrate), characterized by intense roughly granular fluorescence of hepatocyte cytoplasm, bright fluorescence of the first portion of the proximal renal tubules, very specific fluorescence of amine precursor uptake decarboxylation (APUD) stomach cells, and fluorescence of pancreatic Langerhans islets. A link has been established between the detection of AMA-M6 and the uptake of the antidepressant iproniazid. Homberg et al. found that of five patients with AMA-M6, four developed hepatic complications (cytolytic hepatitis or hepatocyte necrosis) while receiving the drug. Following the cessation of iproniazid, the patients’ condition notably improved (6). The fifth patient developed chronic idiopathic autoimmune hemolytic anemia with a very low AMA-M6 titer (1:20), which disappeared within a year.

Several years later, AMA-M6 was shown to be directed against MAO-B (monoamine oxidase B), and to block its enzymatic activity (7). It is noteworthy that iproniazid is a non-specific and irreversible MAO inhibitor, and the author proposes a haptenization mechanism that could be responsible for the formation of autoantibodies directed against the MAO-B – iproniazid complex.

Diagnosis of AMA-M6 is typically achieved through indirect immunofluorescence on rat organs, which reveals the characteristic pattern previously described (6). A patient’s current or past exposure to iproniazid may be an additional diagnostic indicator. Indeed, the literature shows that most AMA-M6 cases have developed under iproniazid therapy. Finally, biological tests can be done to evaluate liver function, with a focus on transaminases. When AMA-M6 has been induced by iproniazid, the symptoms regress once the drug is discontinued (6).

Owing to the side effects of iproniazid, including those on the liver, and all the interactions with other drugs, it is being prescribed less and less, and AMA-M6 cases have become very rare.

Here we present two cases of immunofluorescent AMA-M6-like pattern with atypical clinical presentations diagnosed at the University Hospital of Clermont-Ferrand, France, since patients have never been exposed to iproniazid.

## 2 Case 1

The first case study concerns a 60-year-old man with a history of hypertension, dyslipidemia and arrhythmia, treated with bisoprolol, ramipril, rivaroxaban and pravastatin. His tobacco consumption was estimated at 80 pack-years, but he stopped smoking two months earlier because of a persistent cough. This cough, which was refractory to several antibiotic treatments, was found to be caused by lung adenocarcinoma.

**Figure 1:**
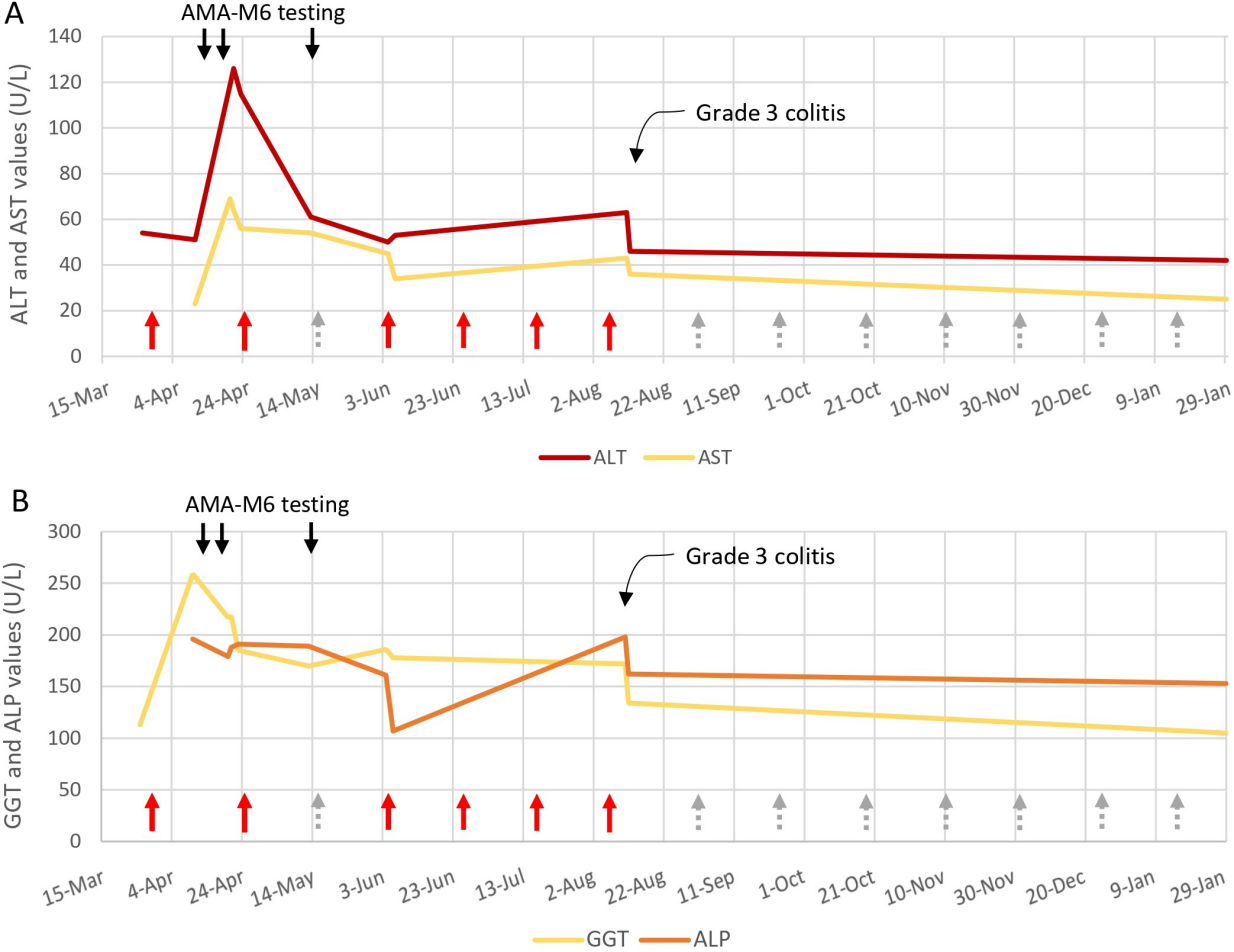
Variation in liver enzymes [**(A)** ALT and AST, **(B)** GGT and ALP] over time and in relation to pembrolizumab injection and AMA-M6-like pattern presence. Solid arrow indicates pembrolizumab injection in combination with chemotherapy. Dotted arrow indicates chemotherapy not associated with pembrolizumab. ALT, alanine aminotransferase; AST, aspartate aminotransferase; GGT, gamma-glutamyltransferase; ALP, alkaline phosphatase; AMA-M6, type 6 antimitochondrial antibody.

**Figure 2:**
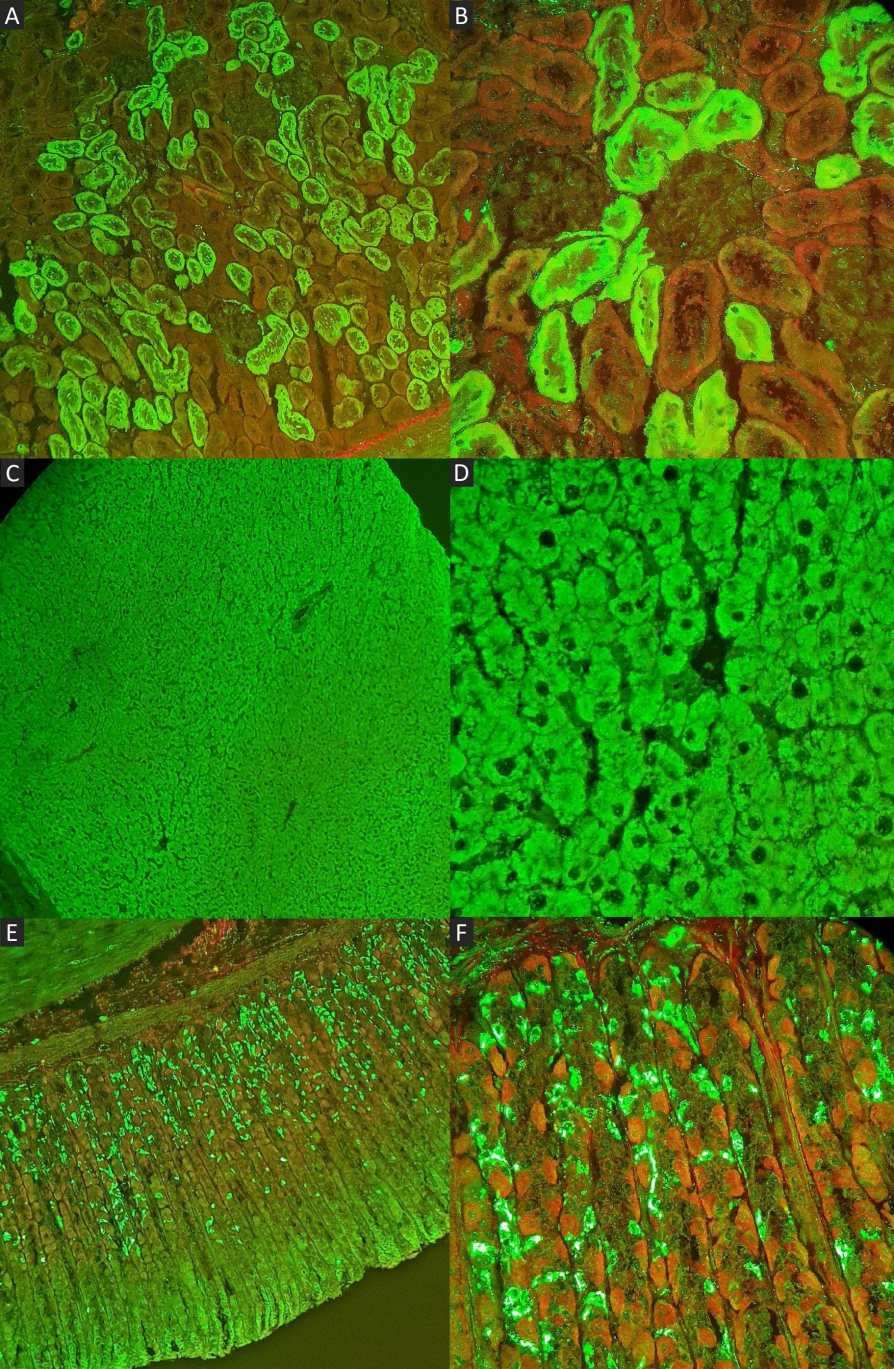
Indirect immunofluorescence patterns observed in the first patient on rat substrates at low [x100, **(A, C, E)**] and high [x400, **(B, D, F)**] magnification are indicative of an AMA-M6-like pattern. **(A, B)** positive fluorescence in the proximal part of the renal tubules. **(C, D)** coarse granular fluorescence in the liver. **(E, F)** fluorescence of the APUD system cells in the stomach APUD, amine precursor uptake and decarboxylation.

Given that 90% of the tumor cells express PD-L1 on their surface, treatment began with a combination of pembrolizumab, carboplatin, and pemetrexed. The treatment was well tolerated, but 19 days after the initial immunotherapy/chemotherapy session, the patient was admitted to the emergency department for severe diarrhea, hypotension, and hyponatremia (125 mmol/L). At that time, liver biochemistry showed cholestasis, which had been present before the start of treatment, and no cytolysis, with alanine aminotransferase (ALT) and aspartate aminotransferase (AST) levels in the normal range. **Figure 1** and **Supplementary Table 1** show the fluctuations in liver markers over time. All biochemical analyses were performed on a Siemens Dimension Vista 1500 automated platform. The following day, a blood sample was collected and sent to the immunology laboratory for further analysis. Test results indicated the absence of anti-nuclear antibodies by indirect immunofluorescence on HEp-2 cells (Inova Diagnostics, San Diego, USA), although the cytoplasm had a homogeneous dense pattern. The results of indirect immunofluorescence on rat organ substrates (MeDiCa, Scimedx Corporation, Dover, USA) are shown in **Figure 2**. An AMA-M6-like pattern was observed, remaining positive up to a dilution of 1:640. The findings were replicated in another sample collected 10 days later, which gave a positive result up to a dilution of 1:2560. No distinctive fluorescence was observed on the islets of Langerhans in primate pancreas sections (Inova Diagnostics, San Diego, USA). All other tests performed to investigate hepatic involvement were negative, including additional patterns observed on triple-substrate slides and immunodot assay (Liver Profile 2, Euroimmun, Lübeck, Germany)—AMA-M2, anti-smooth muscle antibodies (ASMA), anti-Liver Kidney Microsomes (LKM), anti-Liver Cytosol-1 (LC-1), and anti-Soluble Liver Antigen (SLA)—as well as viral serologies (HIV-1 and -2, HBV, HCV, EBV, and CMV). The patient’s medical history was reviewed, and it was confirmed that he had never taken iproniazid.

A second course of chemotherapy treatment was administered. However, hepatitis cytolysis occurred 29 days after the initial injection, with a twofold elevation of ALT and AST levels (**Figure 1**). Quantitative immunoglobulin testing showed a moderate increase in IgG (19.55 g/L; reference range 6.10–16.16 g/L) and IgA (5.26 g/L; reference range 0.85–4.99 g/L) (Optilite, The Binding Site, Birmingham, UK), with an electrophoretic profile consistent with an inflammatory response. The cytolysis was attributed to pembrolizumab, an immunotherapy drug known to cause immune-related adverse events (IRAEs). Accordingly, the third course of chemotherapy was modified, omitting pembrolizumab, and a liver biopsy was scheduled. Following this interruption, ALT and AST levels gradually decreased, allowing the restart of pembrolizumab for the fourth cycle, without a significant rebound. The biopsy results were consistent with regenerative nodular hyperplasia, without evidence of autoimmune hepatitis.

Immunotherapy was maintained for 2 months, during which time no further liver damage occurred. Regrettably, indirect immunofluorescence was not performed on the liver, kidney, and stomach substrate to check for possible negativity of the AMA-M6-like pattern during this period. Grade 3 colitis, treated with corticosteroids, unfortunately prompted discontinuation of pembrolizumab and the pursuit of maintenance therapy with pemetrexed alone. In view of the good clinical and radiological results, pemetrexed was stopped after the 55^th^ cycle. Today the patient is in good health and has not experienced any further liver cytolysis.

## 3 Case 2

The second case study was that of a 70-year-old woman who consulted an internal medicine specialist in 2024 for xerostomia. She had hypothyroidism treated with hormone supplementation. Additionally, she was treated for hypertension with lercanidipine and irbesartan, and for dyslipidemia with simvastatin and ezetimibe.

**Figure 3:**
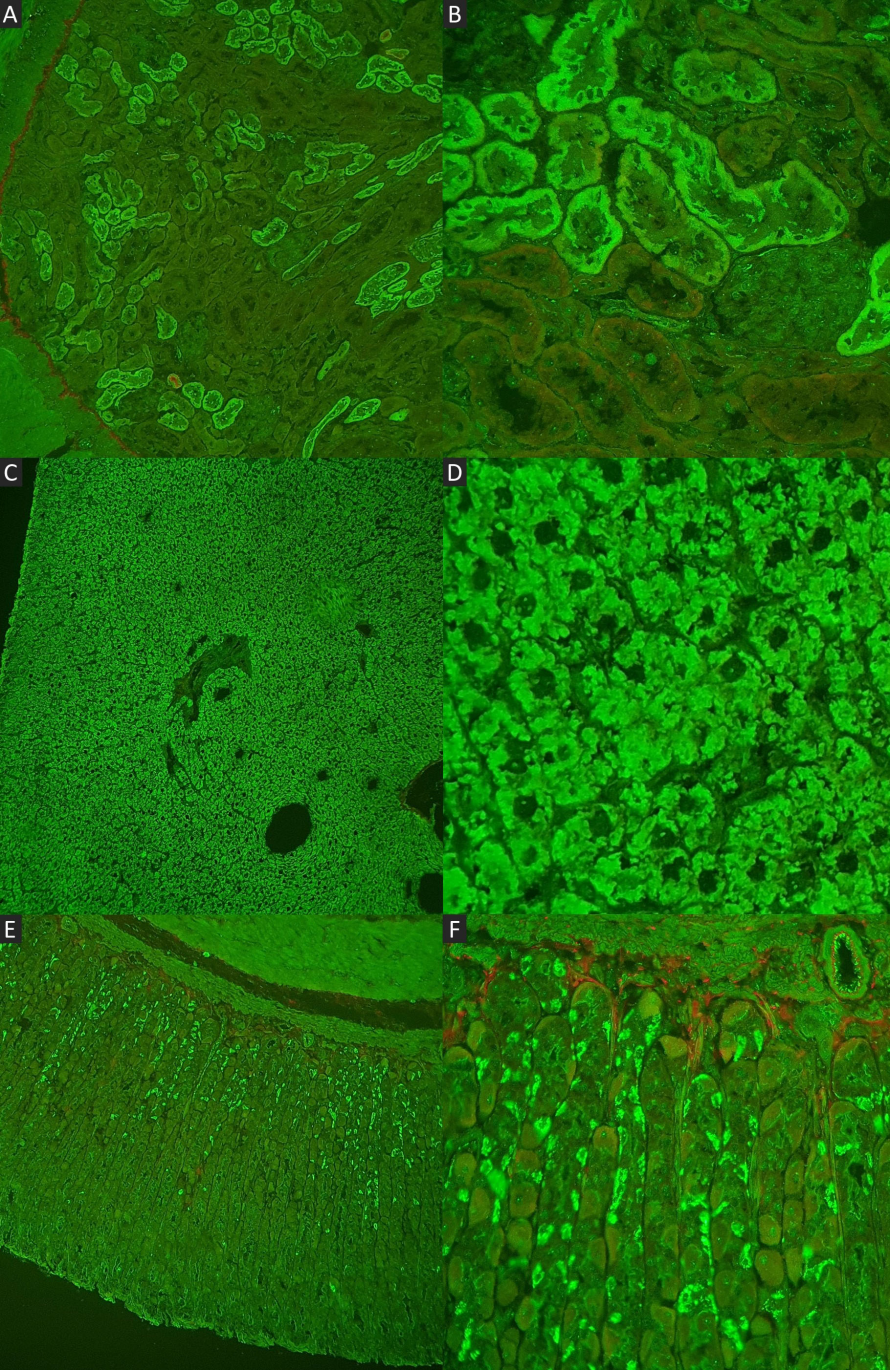
Indirect immunofluorescence patterns observed in the second patient on rat substrates at low [x100, **(A, C, E)**] and high [x400, **(B, D, F)**] magnification are indicative of an AMA-M6-like pattern. **(A, B)** positive fluorescence in the proximal part of the renal tubules. **(C, D)** coarse granular fluorescence in the liver. **(E, F)** fluorescence of the APUD system cells in the stomach. APUD, amine precursor uptake and decarboxylation.

The patient had been reporting a sensation of dry mouth for approximately six months. She also described leg pain which may correspond to myalgia. These symptoms emerged concurrently with a change in lipid-lowering treatment and the initiation of simvastatin. Biologically, creatine kinase levels were in the normal range, as were hepatic enzymes. A more comprehensive investigation was conducted to rule out liver damage. Immunological analysis revealed the absence of antinuclear antibodies (nuclear or cytoplasmic), both by indirect immunofluorescence on HEp-2 cells (Inova Diagnostics, San Diego, USA) and by Luminex technique (absence of autoantibodies directed against dsDNA, chromatin, SS-A52, SS-A60, SS-B, RNP-A, RNP-68, Sm) (Bioplex 2200, Bio-Rad Laboratories, Hercules, USA). However, indirect immunofluorescence analyses on rat organ substrates (MeDiCa, Scimedx Corporation, Dover, USA) revealed the presence of AMA-M6-like pattern, up to a dilution of 1:2560. The images obtained are shown in **Figure 3**. As in the first case, the islets of Langerhans were negative on primate pancreas sections (Inova Diagnostics, San Diego, USA), and no reactivity was detected using an immunodot assay (Liver Profile 2, Euroimmun, Lübeck, Germany), including AMA-M2, ASMA, anti-LKM, anti-LC-1, and anti-SLA. A review of the patient’s medical history confirmed the absence of any previous prescriptions for iproniazid. The physician confirmed the diagnosis of xerostomia and initiated a symptomatic treatment with anetholtrithione.

In this case, the presence of the autoantibody had not caused any liver disorders. However, the patient is now being followed up with liver enzyme tests every six months.

## 4 Additional investigations: assessment of anti–MAO-B autoantibodies

To assess whether the autoantibodies present in the sera of our two patients reacted against MAO-B, as reported in historical cases, two complementary assays were performed; however, neither supported this hypothesis. Competitive inhibition assays using indirect immunofluorescence on triple-substrate slides showed no change in fluorescence intensity following pre-incubation of the sera with a range of MAO-B concentrations. Representative results are presented in the **Supplementary Data** (**Supplementary Figures 1**, **2**). Furthermore, ELISA experiments, performed with various MAO-B concentrations and serum dilutions, did not demonstrate any specific reactivity in either patient (**Supplementary Data**, **Supplementary Figure 3**).

## 5 Discussion

M6-subtype anti-mitochondrial antibody presents a rare immunofluorescent autoantibody pattern first described in patients undergoing treatment with iproniazid, an antidepressant drug. Diagnosis can be made by observing a typical feature visible in indirect fluorescence on rat organ substrates. Positive fluorescence is seen in the APUD stomach cells, the hepatocyte cytoplasm (which has a coarse granular pattern), the initial portion of the proximal renal tubules, and pancreatic islet cells (6). The positivity of APUD cells, also called enterochromaffin cells, is a highly characteristic, almost pathognomonic feature of this autoantibody. These cells are part of the diffuse neuroendocrine system, and secrete a number of hormones including catecholamines and serotonin (8). The fluorescence of these cells is one of the differences in indirect immunofluorescence between the well-known AMA-M2 and AMA-M6 (**Table 1**). The granular hepatic fluorescence is comparable, but the features observed on stomach and renal cells are different. The indirect immunofluorescence aspects observed in our laboratory for the two patients were identical to those previously described in the literature or previously identified in our department and linked to iproniazid consumption. It is nevertheless noteworthy that the fluorescence observed in pancreatic islet cells in rat tissue sections was absent in our analyses on primate tissue sections.

**Table 1:** Indirect immunofluorescence differences between two autoantibodies: AMA-M2 and AMA-M6.

Substrate	AMA-M2	AMA-M6
HEp-2	Cytoplasmic reticular	No characteristic feature
Rat stomach	Parietal cells	APUD cells
Rat kidney	Renal tubules	Proximal renal tubules
Rat liver	Hepatocyte cytoplasm with granular pattern	Hepatocyte cytoplasm with granular pattern

AMA-M2, M2-subtype antimitochondrial antibody; AMA-M6, M6-subtype antimitochondrial antibody; APUD, amine precursor uptake and decarboxylation.

A strong link was found between AMA-M6 and iproniazid with the discovery of the AMA-M6 target (7). The hapten hypothesis and the formation of a novel antigen resulting from the combination of MAO-B and its inhibitor (i.e., iproniazid) may explain the occurrence of AMA-M6 in these patients. However, neither of our patients had ever taken iproniazid. To our knowledge, this is the first description of elevated titers of AMA-M6-like pattern in patients who have never been exposed to iproniazid.

Pembrolizumab is a monoclonal antibody that targets the programmed death-1 (PD-1) protein, a checkpoint molecule involved in the regulation of immune response. This protein blocks the inhibitory interaction between tumor cells and T lymphocytes, thereby enhancing the lymphocyte antitumor activity (9, 10). However, the reactivation of immune functions by a checkpoint inhibitor can also trigger auto-immune diseases. Thus immune-related adverse events occur in 50-72% of patients treated with anti-PD-1 immunotherapy (11, 12). The adverse effects can affect any organ, but are most prevalent in the gut, liver, lungs, skin, and endocrine system (12, 13). Liver adverse effects have been noted in 1-31% of the patients under immunotherapy, depending on the study (13, 14). The spectrum of damage is broad and includes asymptomatic elevation of hepatic enzymes, panlobular hepatitis, portal inflammation, steatosis and steatohepatitis, granuloma, and nodular regenerative hyperplasia (14–16). In our first clinical case, the biopsy findings were consistent with a diagnosis of nodular regenerative hyperplasia, but without evidence of a strict autoimmune origin, as described in patients treated with this checkpoint inhibitor (15). In the light of the previous cases reported, it can be postulated that pembrolizumab may be responsible for the liver damage and the hepatic enzyme elevation in this patient. Besides clinical symptoms seen 19 days following the initial administration of pembrolizumab, the hepatitis cytolysis was observed 10 days later, and AST and ALT levels declined following the cessation of immunotherapy. Based on these data, the RUCAM causality score was calculated at 6, indicating a probable causal relationship; however, a potential contribution of concomitant chemotherapy and other medications cannot be fully excluded. Indeed, carboplatin and, to a lesser extent, pemetrexed have been associated with hepatic adverse effects, including transient elevation in liver enzymes, hepatitis, and hepatocellular injury. Nevertheless, these toxicities are generally mild and self-limited, and more pronounced liver injury has predominantly been reported in the context of combination therapy with immune checkpoint inhibitors (17, 18). The patient was also receiving statin therapy, which is generally well tolerated but has been rarely associated with autoimmune hepatitis, characterized by autoantibody positivity and histological features consistent with autoimmune liver disease (19). However, in patient 1, the resolution of hepatitis despite continued statin exposure makes a contribution of this drug less likely, although a potential role cannot be completely excluded. Thus, the available data suggest a predominant role of pembrolizumab in the liver injury, although other contributing factors cannot be entirely excluded.

A link between pembrolizumab and AMA-M6-like pattern is less well-established. Although the underlying mechanism of pembrolizumab is consistent with the development of autoantibodies, this immunofluorescence pattern is highly uncommon and, to the best of our knowledge, has never been documented previously in this context. One possible hypothesis is a loss of immune tolerance to MAO-B induced by immune checkpoint inhibitor therapy, this protein having been describe as the target of AMA-M6 in iproniazid-treated patients (7). This loss of immune tolerance is now well recognized in patients treated with immune checkpoint inhibitors, as illustrated by cutaneous toxicities associated with the development of anti–BP180 antibodies (20), as well as by cases of myasthenia gravis with positive anti–acetylcholine receptor antibodies (21, 22). However, autoantibodies detected in the sera of both patients did not appear to target MAO-B, as demonstrated by competitive inhibition assays and ELISA. Immunization against MAO-B is thought to result from the binding of iproniazid to the enzyme, leading to the formation of a neoantigen. In the absence of this drug, an alternative mechanism – such as haptenization or another yet unidentified process – may be involved in the development of autoantibodies producing a similar pattern on triple-substrate slides.

In our first case, the AMA-M6-like pattern was identified on three occasions during immunotherapy treatment, with high titers (1:640 to 1:2560), but unfortunately no serum sample was collected after pembrolizumab withdrawal to follow the kinetics of this autoantibody after therapy. A review of the existing literature on AMA-M6 did not reveal any known clinical case linking it to the presence of nodular regenerative hyperplasia. In AMA-M6-associated hepatitis caused by iproniazid, the recurrence of liver damage has consistently been observed following the resumption of the drug (6), in contrast to our findings. In fact, pembrolizumab was reintroduced without liver relapse. It would be interesting to see whether other comparable cases emerge in the future and, if so, what the outcome of such cases are.

Finally, severe diarrhea led to the diagnosis of pembrolizumab-induced grade-3 colitis. This side effect, well-described in trials (12–14), occurred 6 months after starting immunotherapy, the time lapse usually reported (14). Symptoms improved after stopping pembrolizumab, and the drug was not reintroduced. Eventually, clinicians should remain aware of the potential development of autoimmune diseases in patients treated with immune checkpoint inhibitors and investigate them in the presence of biological or clinical abnormalities in order to provide optimal patient care.

In the second case, the factors determining the appearance of AMA-M6-like pattern are more elusive. The only symptom reported was xerostomia, and transaminase levels remained in the normal range. As in the first case, the patient was receiving statin therapy, although no evidence of hepatitis or other autoimmune manifestations previously associated with such treatment was observed. Furthermore, xerostomia has not been reported in this context. Autoantibodies are frequently detected prior to the onset of clinical symptoms, as observed in systemic lupus erythematosus or primary biliary cholangitis (5, 23). Careful long-term follow-up therefore appears essential to allow early detection of potential liver injury and timely clinical intervention.

## 6 Conclusion

In conclusion, we describe two newly diagnosed cases of M6-like-subtype antimitochondrial antibody. Although it is commonly assumed that AMA-M6 is associated with the use of iproniazid, this was not the case here. One case was reported in a context of lung cancer with a treatment regimen including pembrolizumab. Liver cytolysis, considered as a hepatic IRAE, was documented one month following the initial immunotherapy injection. Liver biopsy results showed regenerative nodular hyperplasia, a feature that has been described on several occasions following the administration of pembrolizumab. A link between pembrolizumab and AMA-M6-like pattern was more challenging to ascertain, as this association had never been described previously. However, it would not be unexpected, given the mechanism of action of the drug, the time course, and the presentation of the symptoms. The second case study was that of a woman treated for hypertension and dyslipidemia with drugs that are widely prescribed and not previously associated with the indirect immunofluorescence features observed here. The patient reported only a sensation of dry mouth, with no evidence of liver damage. A monitoring program is currently in place to track any hepatic injury.

The identification of autoantibodies producing an AMA-M6-like pattern in diverse clinical contexts challenges their traditional association with iproniazid, particularly as these antibodies do not appear to target MAO-B, contrary to earlier reports. While the precise antigenic target of these autoantibodies remains to be elucidated, their presence does not appear to be specifically associated with liver injury. Recognizing the rare and distinctive immunofluorescence patterns of these autoantibodies is therefore essential to avoid misinterpretation. Awareness of these atypical profiles is crucial for accurate diagnosis and appropriate clinical management and follow-up.

## 7 Materials and methods

### 7.1 Indirect immunofluorescence assay

AMA-M6-like pattern were detected by indirect immunofluorescence using routine laboratory procedures on triple-substrate slides composed of rat stomach, liver, and kidney (MeDiCa, Scimedx Corporation, Dover, USA), according to the manufacturer’s instructions. Serum samples were diluted in phosphate-buffered saline (PBS) at 1:40, 1:160, 1:640, and 1:2560. Forty microliters of each diluted serum sample were applied to the slides and incubated for 30 minutes at room temperature. After washing, 40 µL of fluorescein-labeled anti-human Ig conjugate were added, and the slides were incubated for 30 minutes at room temperature. Following a final wash, slides were observed under a fluorescence microscope. Positive controls (antinuclear antibodies, AMA-M2, anti-smooth muscle antibodies, or anti-parietal cell antibodies) and negative controls (PBS) were systematically included.

### 7.2 Competitive inhibition experiments

Recombinant MAO-B (Invitrogen, Fisher Scientific, Illkirch-Graffenstaden, France) was pre-incubated with diluted patient sera (1:640) in PBS at final concentrations ranging from 0.1 to 20 µg/mL (0.1, 0.2, 1, 2, 10, and 20 µg/mL). Incubations were performed for 1 or 2 hours at room temperature or 37 °C. Subsequently, 50 µL of each mixture was applied to triple-substrate slides containing rat stomach, liver, and kidney sections (Kallestad™; Bio-Rad Laboratories, Hercules, CA, USA). The remainder of the procedure was performed as described above.

### 7.3 ELISA

Coating solutions were prepared by diluting recombinant MAO-B in 50 mM carbonate buffer (pH 9.4; Thermo Fisher Scientific, Waltham, MA, USA) to final concentrations of 1, 2, and 5 µg/mL. Ninety-six–well plates were coated overnight at 4 °C. Plates were then washed (ELISA Wash Buffer; Thermo Fisher Scientific) and blocked for 2 hours at room temperature using assay buffer (Thermo Fisher Scientific).

Sera were diluted in Assay buffer (1:1,000 to 1:100,000) and incubated for 90 minutes at room temperature with continuous shaking. After washing, HRP-conjugated goat anti-human IgG (Thermo Fisher Scientific) was added at 20 ng/mL and incubated for 2 hours at room temperature with continuous shaking. Following washing steps, tetramethylbenzidine (TMB) substrate solution (Thermo Fisher Scientific) was added and incubated for 30 minutes at room temperature in the dark before addition of the stop solution (Thermo Fisher Scientific). Absorbance was measured at 450 nm immediately thereafter.

## Data Availability

The original contributions presented in the study are included in the article/**Supplementary Material**. Further inquiries can be directed to the corresponding author.

## Supplementary material

Supplementary Table 1 Liver enzyme levels over time according to pembrolizumab administration and AMA-M6-like pattern.

Supplementary Figure 1 No reduction in fluorescence intensity was observed in patient 1, as assessed by indirect immunofluorescence on rat substrate at high magnification (×400) using a serum dilution of 1:640 **(A)**, and following 2-hour incubation with MAO-B at final concentrations of 0.2 µg/mL **(B)**, 2 µg/mL **(C)**, and 20 µg/mL **(D)**.

Supplementary Figure 2 No reduction in fluorescence intensity was observed in patient 2, as assessed by indirect immunofluorescence on rat substrate at high magnification (×400) using a serum dilution of 1:640 **(A)**, and following 2-hour incubation with MAO-B at final concentrations of 0.2 µg/mL **(B)**, 2 µg/mL **(C)**, and 20 µg/mL **(D)**.

Supplementary Figure 3 no specific antibody binding to MAO-B was detected by ELISA in sera from the two patients tested, in comparison with a healthy control serum. Sera were diluted 1:50,000 and 1:100,000 and incubated on uncoated plates or plates coated with MAO-B at 1 or 5 µg/mL. OD_450, optical density at 450 nm.
