# Supplementary material for: Case Report: Two atypical cases of M6-like antimitochondrial antibody pattern unlinked to iproniazid
Source: Front Immunol. 2026 May 29;17:1675688. doi: 10.3389/fimmu.2026.1675688 (PMC13260532; doi:10.3389/fimmu.2026.1675688)
Supplement: Supplementary Table 1 — Liver enzyme levels over time according to pembrolizumab administration and AMA-M6-like pattern. [file Table1.pdf]

Table SI: Liver enzyme levels over time according to pembrolizumab administration and AMA-M6-like pattern.
ALT: alanine aminotransferase, AST: aspartate aminotransferase, GGT: gamma-glutamyltransferase, ALP: alkaline phosphatase, AMA-M6: type 6 antimitochondrial antibody.
"/": not available; "X": pembrolizumab administration.

**Day**	**ALT (U/L)**	**AST (U/L)**	**GGT (U/L)**	**ALP (U/L)**	**Pembrolizumab**	**Indirect immunofluorescence**
26-Mar	54	/	113	/	X	
10-Apr	51	23	259	196		
11-Apr	/	/	/	/		AMA-M6-like pattern detection
20-Apr	119	69	217	179		
21-Apr	126	64	218	188		AMA-M6-like pattern confirmation
23-Apr	115	56	185	191	X	
13-May	61	54	170	189		
14-May	/	/	/	/		AMA-M6-like pattern confirmation
4-Jun	50	45	186	161	X	
6-Jun	53	34	178	107		
25-Jun	/	/	/	/	X	
16-Jul	/	/	/	/	X	
6-Aug	/	/	/	/	X	
11-Aug	63	43	172	198		
12-Aug	46	36	134	162		
29-Jan	42	25	105	153		
