# Supplementary material for: Case Report: Two atypical cases of M6-like antimitochondrial antibody pattern unlinked to iproniazid
Source: Front Immunol. 2026 May 29;17:1675688. doi: 10.3389/fimmu.2026.1675688 (PMC13260532; doi:10.3389/fimmu.2026.1675688)
Supplement: Supplementary Figure 1 — No reduction in fluorescence intensity was observed in patient 1, as assessed by indirect immunofluorescence on rat substrate at high magnification (×400) using a serum dilution of 1:640 (A), and following 2-hour incubation with MAO-B at final concentrations of 0.2 µg/mL (B), 2 µg/mL (C), and 20 µg/mL (D). [file Image1.pdf]

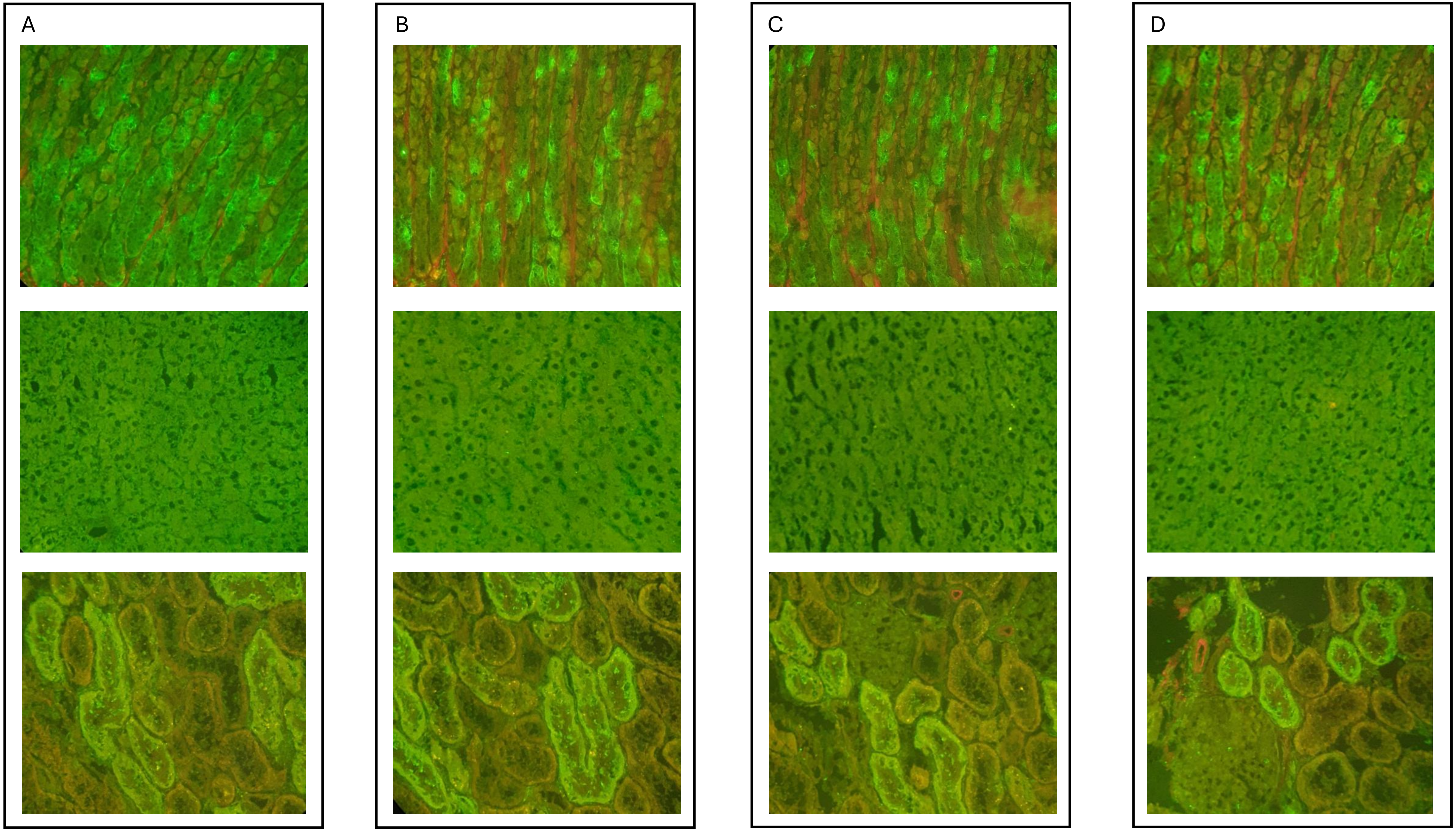


Figure S1: No reduction in fluorescence intensity was observed in patient 1, as assessed by indirect immunofluorescence on rat substrate at high magnification (×400) using a serum dilution of 1:640 (A), and following 2-hour incubation with MAO-B at final concentrations of 0.2 µg/mL (B), 2 µg/mL (C), and 20 µg/mL (D).

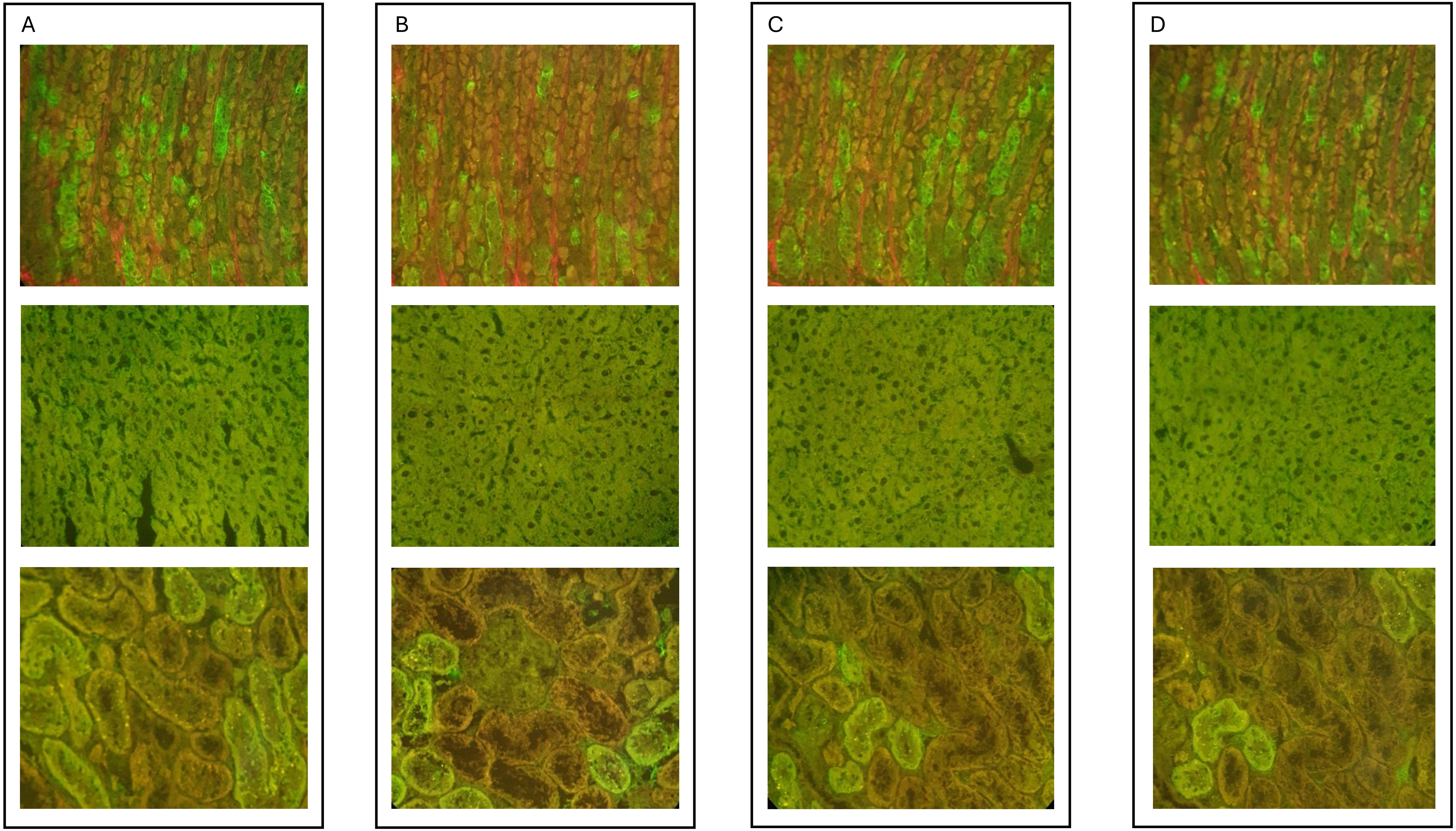


Figure S2: No reduction in fluorescence intensity was observed in patient 2, as assessed by indirect immunofluorescence on rat substrate at high magnification (×400) using a serum dilution of 1:640 (A), and following 2-hour incubation with MAO-B at final concentrations of 0.2 µg/mL (B), 2 µg/mL (C), and 20 µg/mL (D).

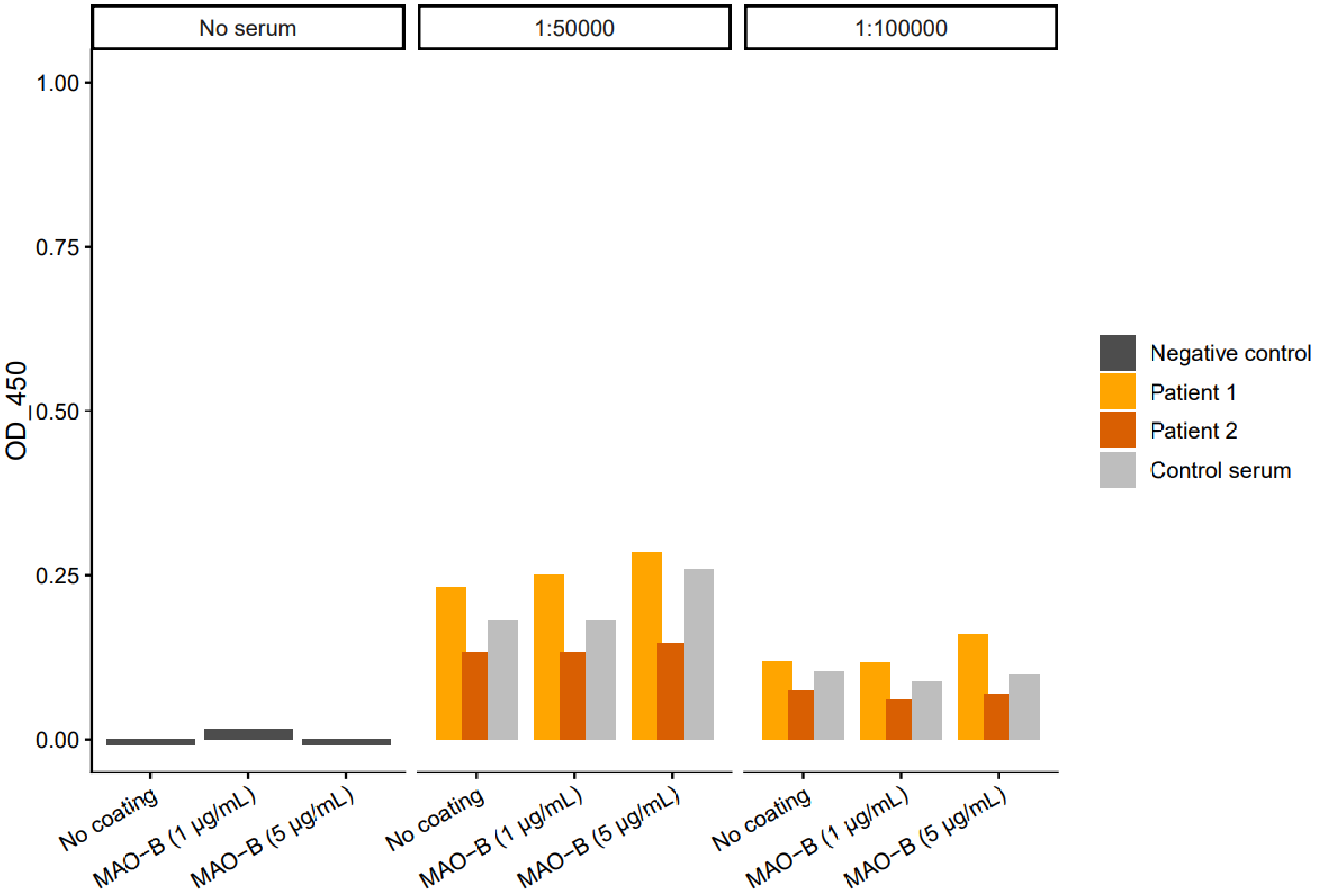


Figure S3: No specific antibody binding to MAO-B was detected by ELISA in sera from the two patients tested, in comparison with a healthy control serum. Sera were diluted 1:50,000 and 1:100,000 and incubated on uncoated plates or plates coated with MAO-B at 1 or 5 µg/mL. OD_450: optical density at 450 nm.
